# Supplementary material for: LC-MS-Based Metabolomics for the Chemosystematics of Kenyan Dodonaea viscosa Jacq (Sapindaceae) Populations
Source: Molecules. 2020 Sep 10;25(18):4130. doi: 10.3390/molecules25184130 (PMC7570515; doi:10.3390/molecules25184130)
Supplement: Supplementary file 1 [file molecules-25-04130-s001.pdf]

SUPPLEMENTARY MATERIAL

LC-MS- Based Metabolomics for the Chemosystematics of Kenyan *Dodonaea viscosa* Jacq (Sapindaceae)

Magrate M. Kaigongi[1,2*], Catherine W. Lukhoba[1], Purity J. Ochieng[3] Malcolm Taylor[4] , Abiy Yenesew[3], Nokwanda P. Makunga[5*]

*kaigongim@gmail.com (M.M.K.); makunga@sun.ac.za (N.P.M

Contents

Abstract......2
1.1.General characteristics of the isolated compounds 1-4 3
1.2.NMR and MS Spectra of the isolated compounds 1-4......7
1.2.1.Dodonic acid (1)......7
1.2.2.5,7,4',5'-Tetrahydroxy-3,6,2' –trimethoxyflavone (2) 10
1.2.3. Hautriwaic acid lactone (3) 12
1.2.4. Catechin (4)......15
1.3.Random permutation and k-fold cross-validation of discriminant chemicals for Kenyan *D. viscosa*......18
References......20

Abstract.

Dodonaea viscosa Jacq (Sapindaceae) is a medicinal plant with a world-wide distribution. The species has undergone enormous taxonomic changes which causes confusion amongst plant users. In Kenya for example, two varieties are known to exist based on morphology; *D. viscosa* var *viscosa* along the coast, and *D. viscosa* var *angustifolia* in the Kenyan inland; these two taxa are recognised as distinct species in some reports. This prompted us to apply metabolomics to understand the relationship among naturally occurring populations of *D. viscosa* in Kenya, and to identify compounds that can assist in taxonomic delineation of the different varieties of *D. viscosa* from different parts of Kenya. The phytochemical variability of Kenyan *D. viscosa* var. *angustifolia* populations collected from four different geographical regions (Nanyuki, Machakos, Nairobi, Narok) and one coastal *D. viscosa* var *viscosa* (the Gazi) were analysed by LC-MS using a metabolomics-driven approach. Four known compounds, two diterpenoids (dodonic acid (**1**), hautriwaic acid lactone (**3**) and two flavonoids [5,7,4',5'-tetrahydroxy-3,6,2'-trimethoxyflavone (**2**) and catechin (**4**) were isolated and purified from the Gazi, coastal collection. The presence of these compounds and their relative abundance in other populations was determined by LC-MS analyses. The use of multivariate statistical analyses of LC-MS data allowed the visualization of the patterns of variation and identification of additional compounds. Eleven discriminant compounds responsible for separating chemometric clusters were tentatively identified. The clustering pattern of the five populations of *D. viscosa* suggested that the metabolite profiles were influenced by geo-environmental conditions and did not support the current classification of *D. viscosa* based on morphology. This study disputes the current classification of *D. viscosa* in Kenya and recommends revision using tools such as molecular phylogenetics. In an antimicrobial assay, hautriwaic acid lactone (**3**) and catechin (**4**) were the most active compounds followed by the extract from coastal (Gazi) population.

Keywords: African traditional medicine; antimicrobial activity; chemosystematics; Hopbush; metabolomics; natural products chemistry; phenolics; terpenoids

1.1. General characteristics of the isolated compounds 1-4

Dodonic acid (**1**) [1,2] white powder with molecular formula $C_{20}H_{28}O_4$ based on electrospray ionization mass spectrometry (ESIMS)([M-H] $^{-}$*m/z* 331.1910). 5,7,4',5'-Tetrahydroxy-3,6,2' –trimethoxyflavone (**2**) [3] yellow amorphous solid, $C_{18}H_{16}O_9$, ESIMS ([M-H] $^{-}$*m/z* 375.0723). Hautriwaic acid lactone (**3**) [1] $C_{20}H_{26}O_3$, ESIMS ([M-H] $^{-}$*m/z* 313.2361) white amorphous solid active under UV (254 nm). Catechin (**4**) [4] brown solid active under UV (254-366 nm) with ESIMS of ([M-H]$^{-}$ *m/z* 289.0719), $C_{15}H_{14}O_6$.

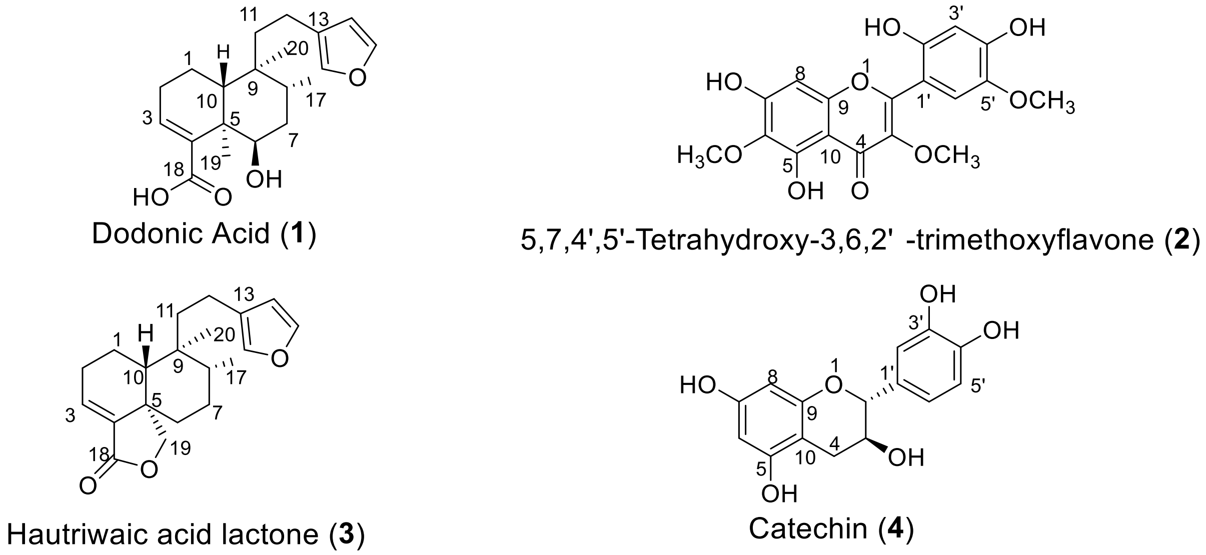

Figure S1. Chemical structures of compounds **1- 4** isolated from coastal plants of *D. viscosa*

1.2. Spectroscopic data for compounds 1-4 isolated from *D. viscosa*

Table S1: ^{1}H (500 MHz), ^{13}C (125 MHz) NMR data along with HMBC correlations (in CD_2Cl_2) for dodonic acid **(1)**

**Position**	**δc**	**δH (m, J in Hz)**	**HMBC**
1	17.2	1.74 (m)	
2	27.5	2.35 (m)	
3	143.9	7.25 (m)	
4	140.2		
5	44.7		
6	74.6	3.77 (dd, 4.9, 10.8)	
7	35.8	1.67 (m)	
8	33.6	1.77 (m)	C-9
9	38.7		
10	45.7	1.42 (m)	C-2, C-4, C-9
11	38.6	1.66 (m)	
12	17.2	1.77 (m)	C-9
13	125.3		
14	138.4	7.26 (br s)	C-13, C-15, C-16
15	142.6	7.38 (br s)	C-13
16	110.9	6.32 (br s)	C-13, C-14, C-15
17	15.3	0.9 (d, 6.6)	C-7, C-9
18	173.1		
19	16.3	1.26 (s)	C-3,C-6, C-10
20	17.8	0.8 (s)	C-8, C-10, C-11

TableS2: ^{1}H (500 MHz), ^{13}C (125 MHz) NMR data along with HMBC correlations (in CD_2Cl_2) for 5,7,4',5'-tetrahydroxy-3,6,2'–trimethoxyflavone (**2**)

**Position**	**δc**	**δH (m)**	**HMBC**
2	155.9		
3	136.4		
4	177.8		
5	151.2		
6	130.2		
7	155.3		
8	93.3	6.60 (s)	C-6, C-7, C-9, C-10
9	152.8		
10	106.0		
1’	109.8		
2’	151.8		
3’	102.2	6.66	C-1’, C-4’, C-5’
4’	150.2		
5’	139.9		

6’	113.3	7.26 (s)	C-2, C-5’
2 ’-OMe	56.15	3.98	C-2’
3-OMe	61.90	3.88	C-3
6-OMe	60.73	4.05	C-6
OH-4’		7.84 (s)	
OH-7		6.67 (s)	
OH-5		12.74 (s)	

Table S3: ^{1}H (500 MHz), ^{13}C (125 MHz) NMR data along with HMBC correlations (in $CDCl_3$) for hautriwaic acid lactone (**3**)

**Position**	**δc**	**δH**	**HMBC**
1	17.9	1.65 (m)	
		1.47 (m)	
2	27.6	2.19 (m)	
3	135.9	6.68 (m)	C-4, C-5, C-18
4	141.5		
5	42		
6	34.5	1.42 (m)	
7	26.6	1.68 (m)	
		1.38 (m)	
8	16.7		
9	19.5		
10	45.7	1.14 (m)	
11	38.6	1.48 (m)	
12	17.2	2.43 (m)	
13	125.3		
14	138.5	7.26 (s)	C-13, C-16, C-15
15	142.7	7.37	
16	110.9	6.31 (br s)	
17	15.3	0.88 (d, 6.6)	
18	169.6		
19	65.1	4.12 (d, 4.2)	C-4, C-5, C-6, C-10, C-18
		3.72 (d, 3.8)	C-4, C-5, C-6, C-10
20	17.3	0.78 (s)	C-17, C-19

Table S4: ^{1}H (500 MHz), ^{13}C (125 MHz) NMR data along with HMBC correlations (in CD_3OD) for catechin (**4**)

**Position**	**δc**	**δ_H (m, *J* in Hz)**	**HMBC**
2	82.9	4.57 (d, 7.5)	C-3, C-1′, C-2′, 6 ′
3	68.8	3.98 (td, 5.4, 7.8)	
4	28.5	2.51 (dd, 8.1, 16.1)	C-2, C-3, C-5, C-9, C-10
5	157.6		
6	96.4	4.93 (d, 2.3)	C-8, C-10
7	157.9		
8	95.6	5.86 (d, 2.3)	C-6, C-10
9	156.9		
10	100.9		
1′	132.3		
2′	115.3	6.84 (d, 2.0)	C-2, C-4′ C-6′
3′	146.2		
4′	146.3		
5′	116.1	6.76 (d, 8.1)	C-2, C-1′ C-3′
6′	120.0	6.72 (dd, 2.0, 8.2)	C-2, C-2′, C-6′

1.2.NMR and MS Spectra of the isolated compounds 1-4

1.2.1. Dodonic acid (1)

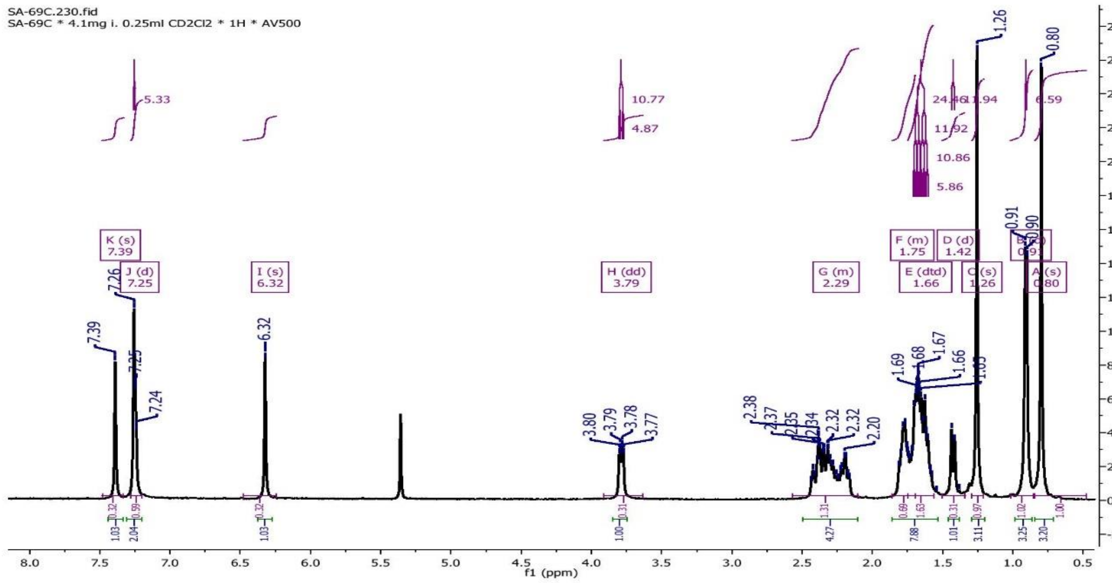

Figure S2. The ^{1}H NMR spectrum of dodonic acid (**1**) observed at 500 MHz in CD_2Cl_2 solution at 25 °C. Assignment is provided in Table S1.

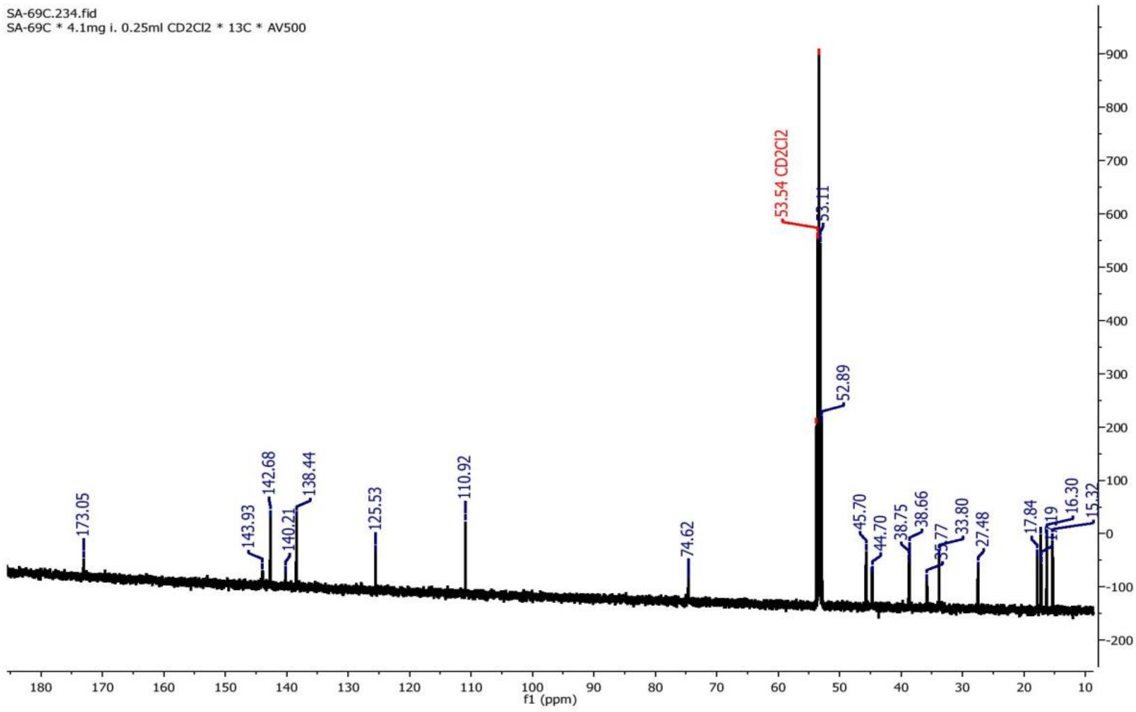

Figure S3. The ^{13}C NMR spectrum of dodonic acid (**1**) observed at 500 MHz in CD_2Cl_2 solution at 25 °C. Assignment is provided in Table S1.

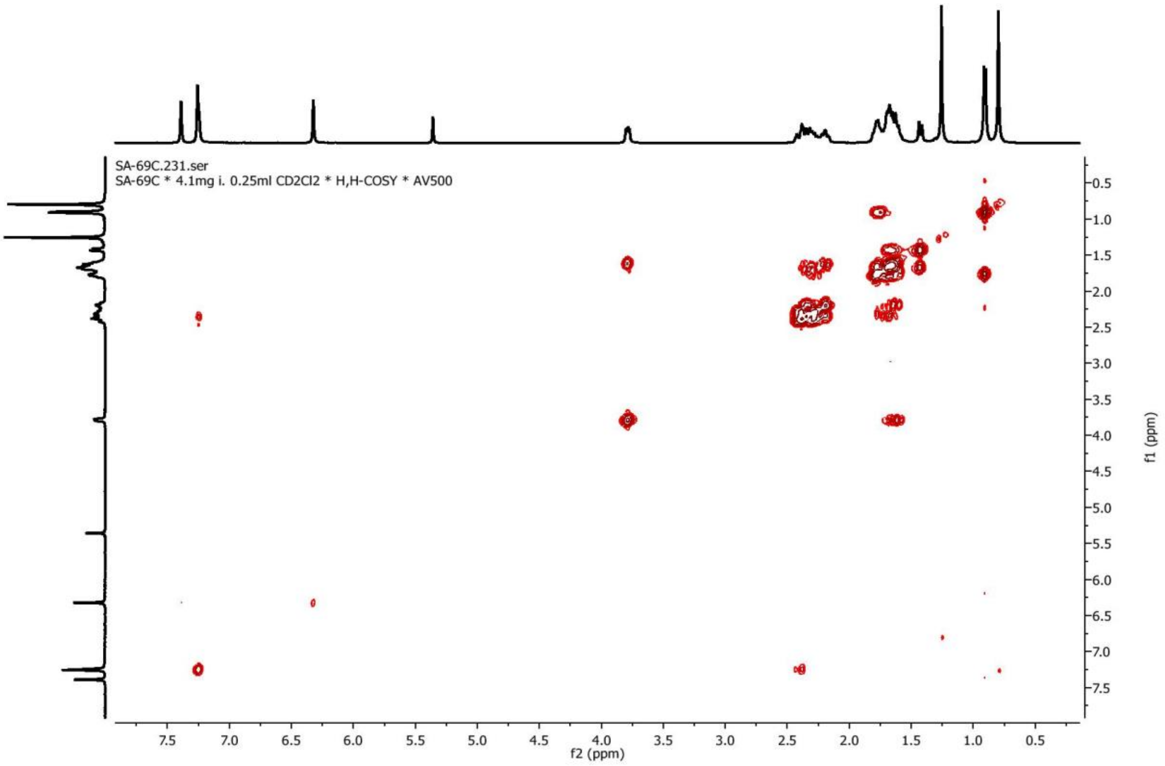

Figure S4. The ^{1}H-^{1}H COSY NMR spectrum of dodonic acid (**1**) observed at 500 MHz in CD_2Cl_2 solution at 25 °C.

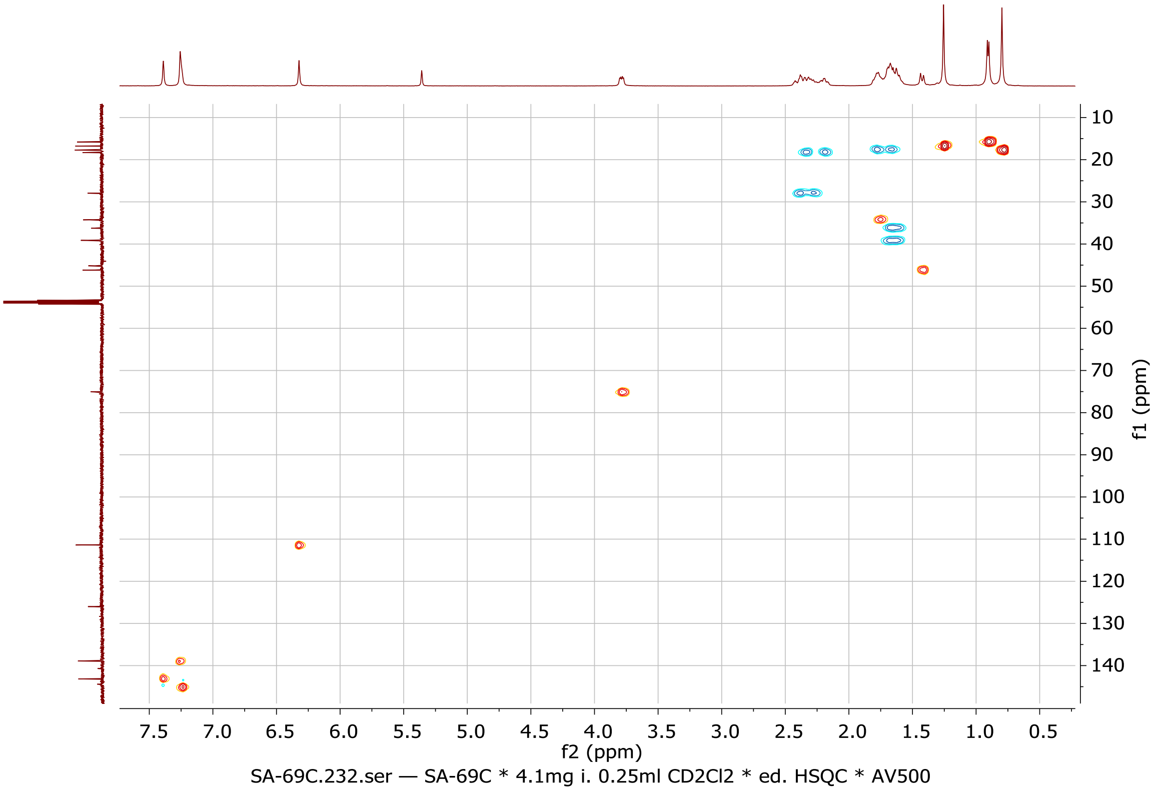

Figure S5. The ^{1}H-^{13}C HSQC NMR spectrum of dodonic acid (**1**) observed at 500 MHz in CD_2Cl_2 solution at 25 °C.

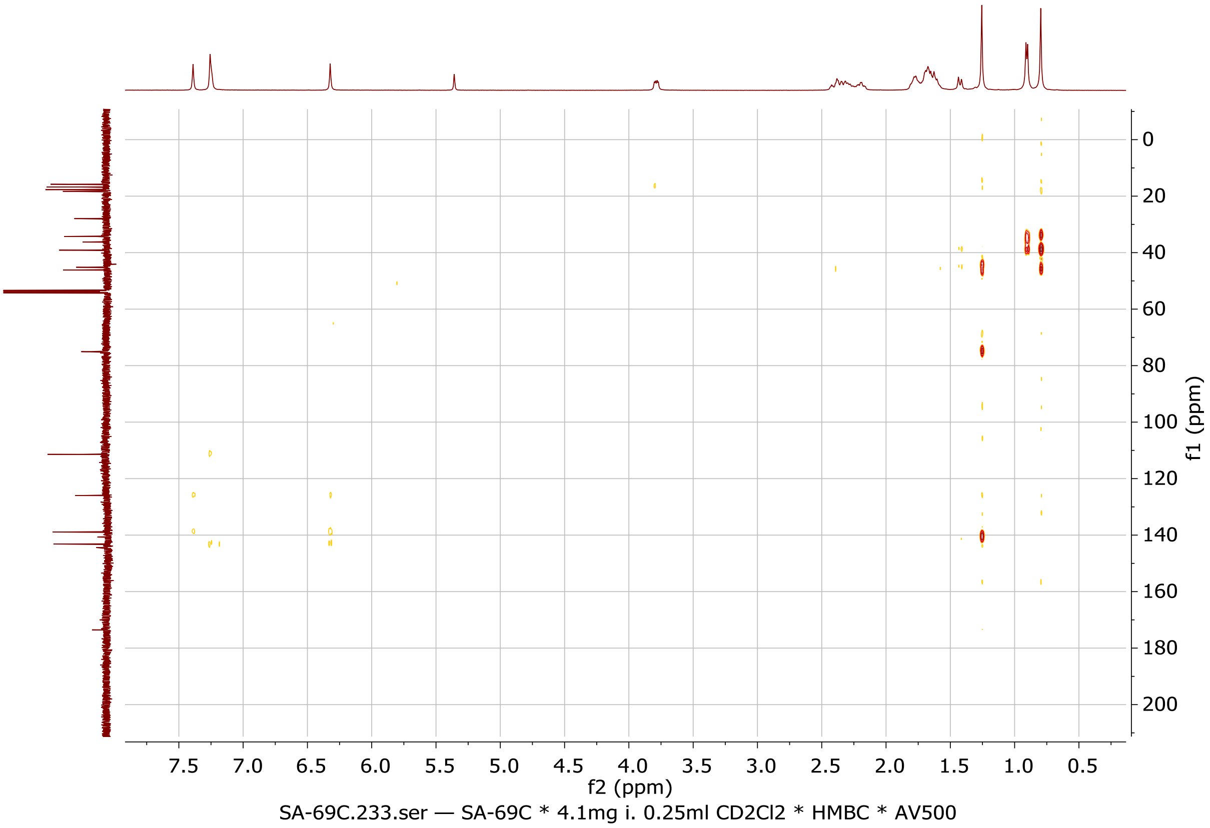

Figure S6. The ^{1}H-^{13}C HMBC NMR spectrum of dodonic acid (**1**) observed at 500 MHz in CD_2Cl_2 solution at 25 °C. Assignment is provided in Table S1.

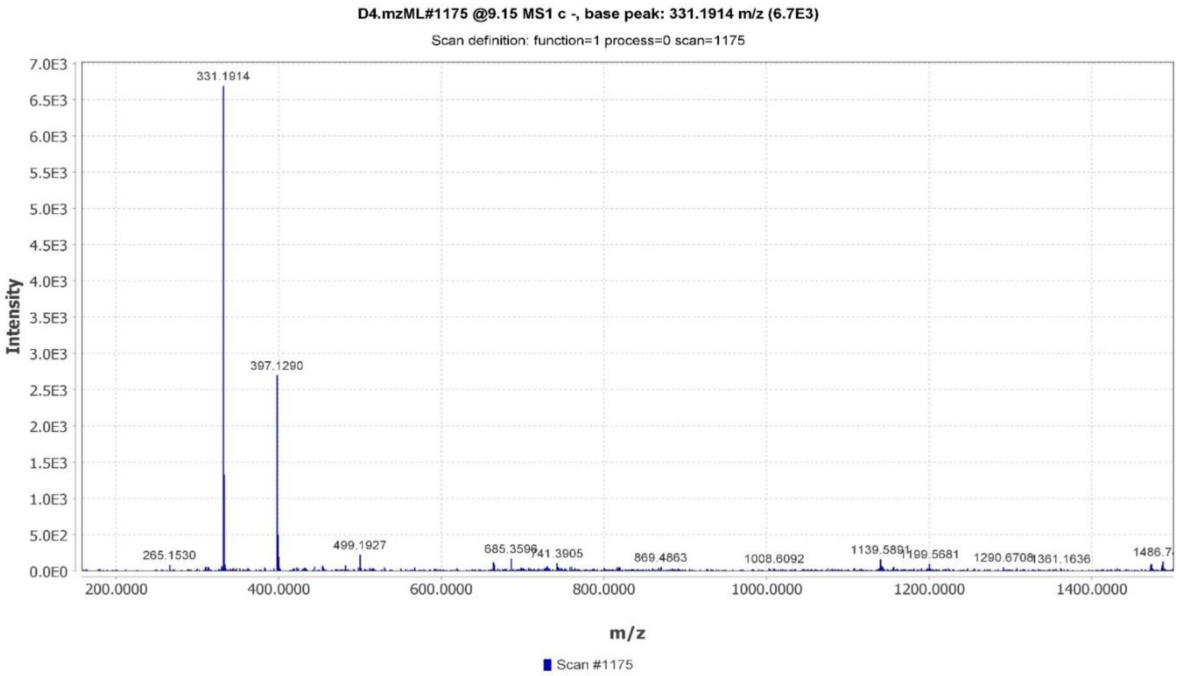

Figure S7. ESIMS spectrum for dodonic acid (**1**)

1.2.2. 5,7,4',5'-Tetrahydroxy-3,6,2' –trimethoxyflavone (2)

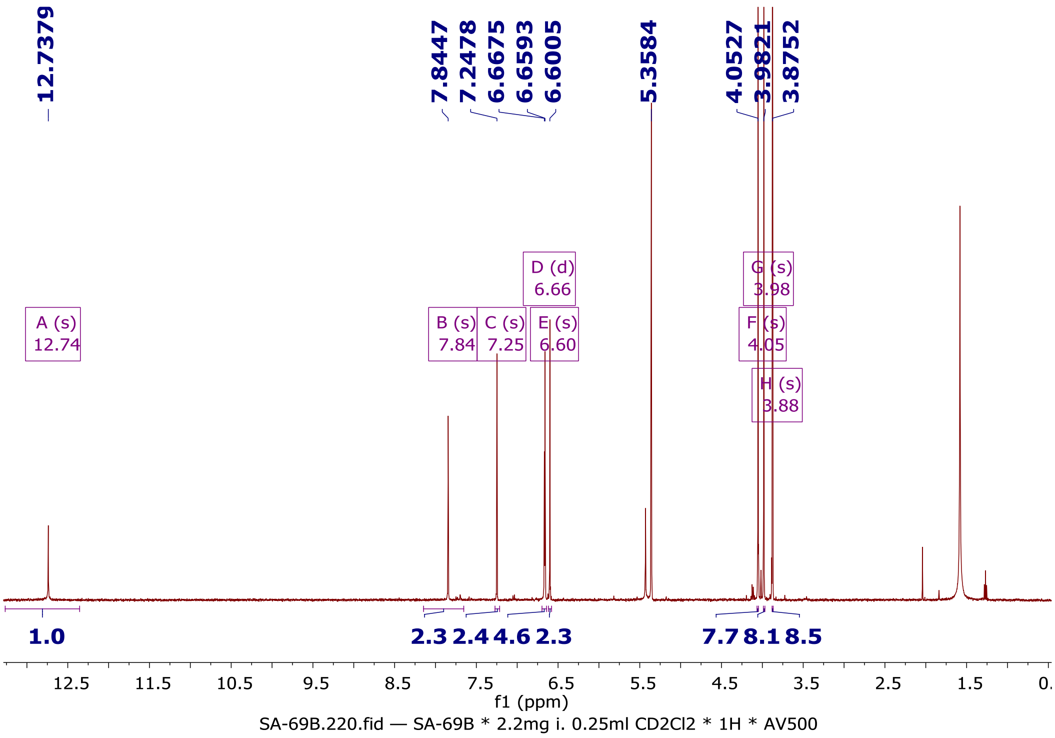

Figure S8. The ^{1}H NMR spectrum of 5, 7, 4', 5' -tetrahydroxy-3, 6, 2’ -trimethoxyflavone (**2**) observed at 500 MHz in CD_2Cl_2 solution at 25 °C. Assignment is provided in Table S2.

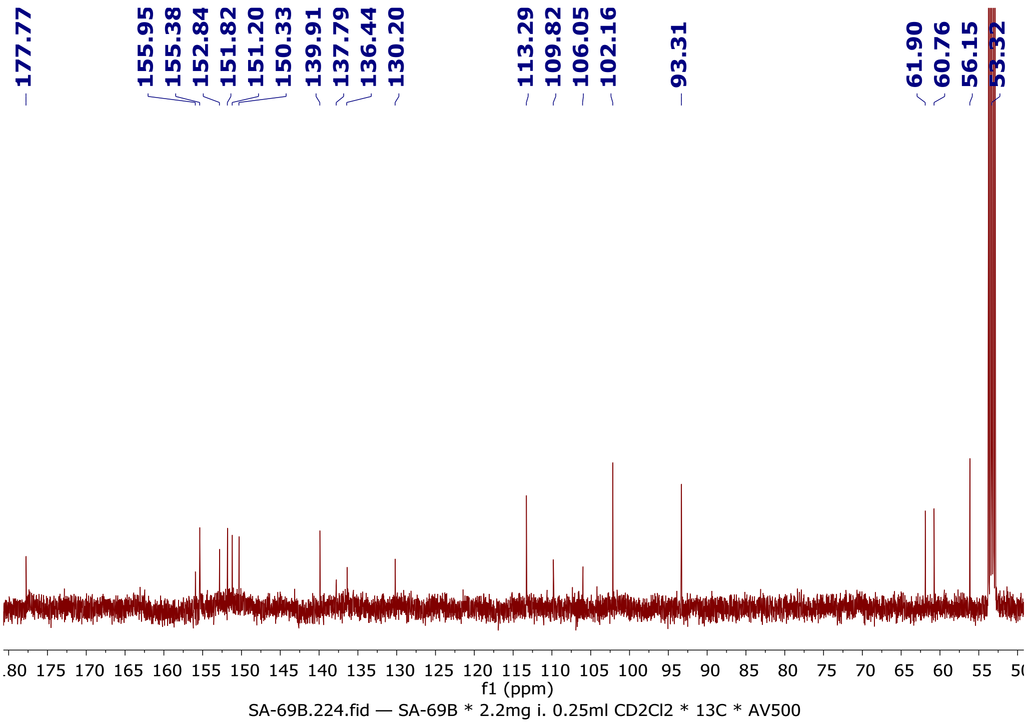

Figure S9. ^{13}C NMR spectrum of 5,7,4',5'-tetrahydroxy-3,6,2’-trimethoxyflavone (**2**) observed at 500 MHz in CD_2Cl_2 solution at 25 °C. Assignment is provided in Table S2.

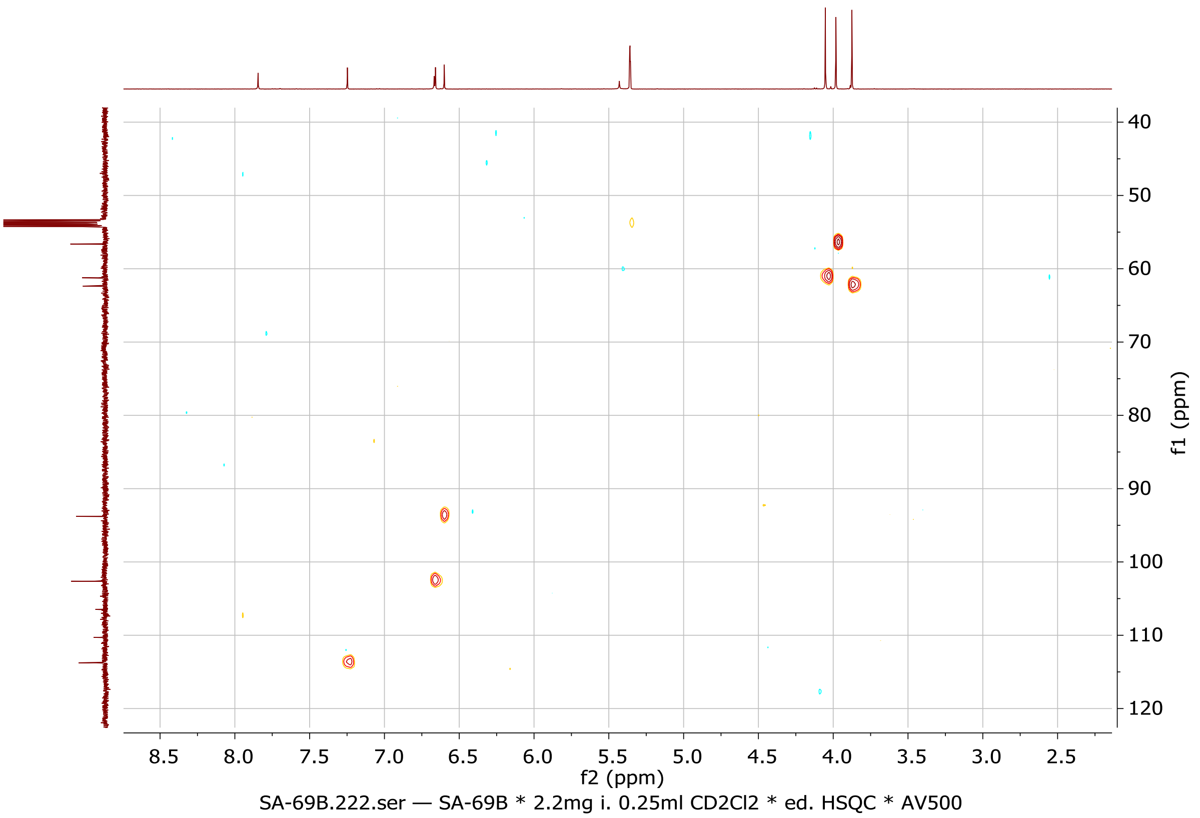

Figure S10. ^{1}H- ^{13}C HSQC spectrum of 5,7,4',5'-tetrahydroxy-3,6,2'-trimethoxyflavone (**2**) observed at 500 MHz in CD_2Cl_2 solution at 25 °C.

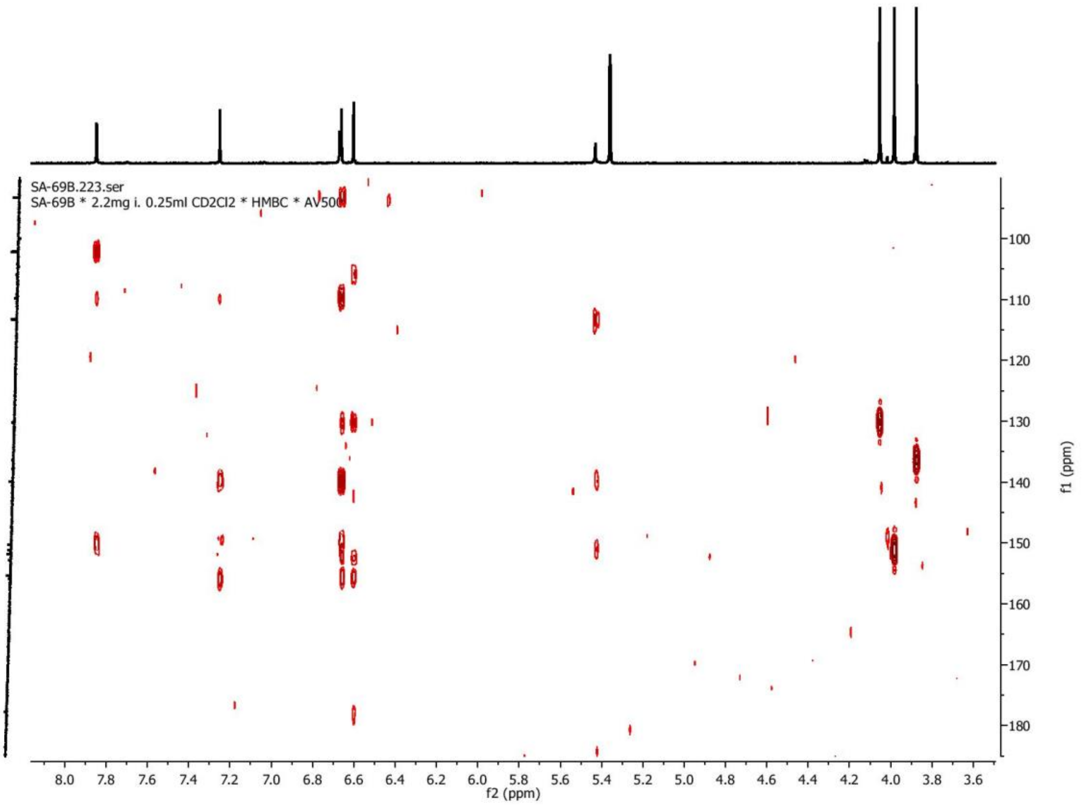

Figure S11. ^{1}H- ^{13}C HMBC spectrum of 5,7,4',5'-tetrahydroxy-3,6,2'-trimethoxyflavone (**2**) observed at 500 MHz in CD_2Cl_2 solution at 25 °C. Assignment is provided in Table S2.

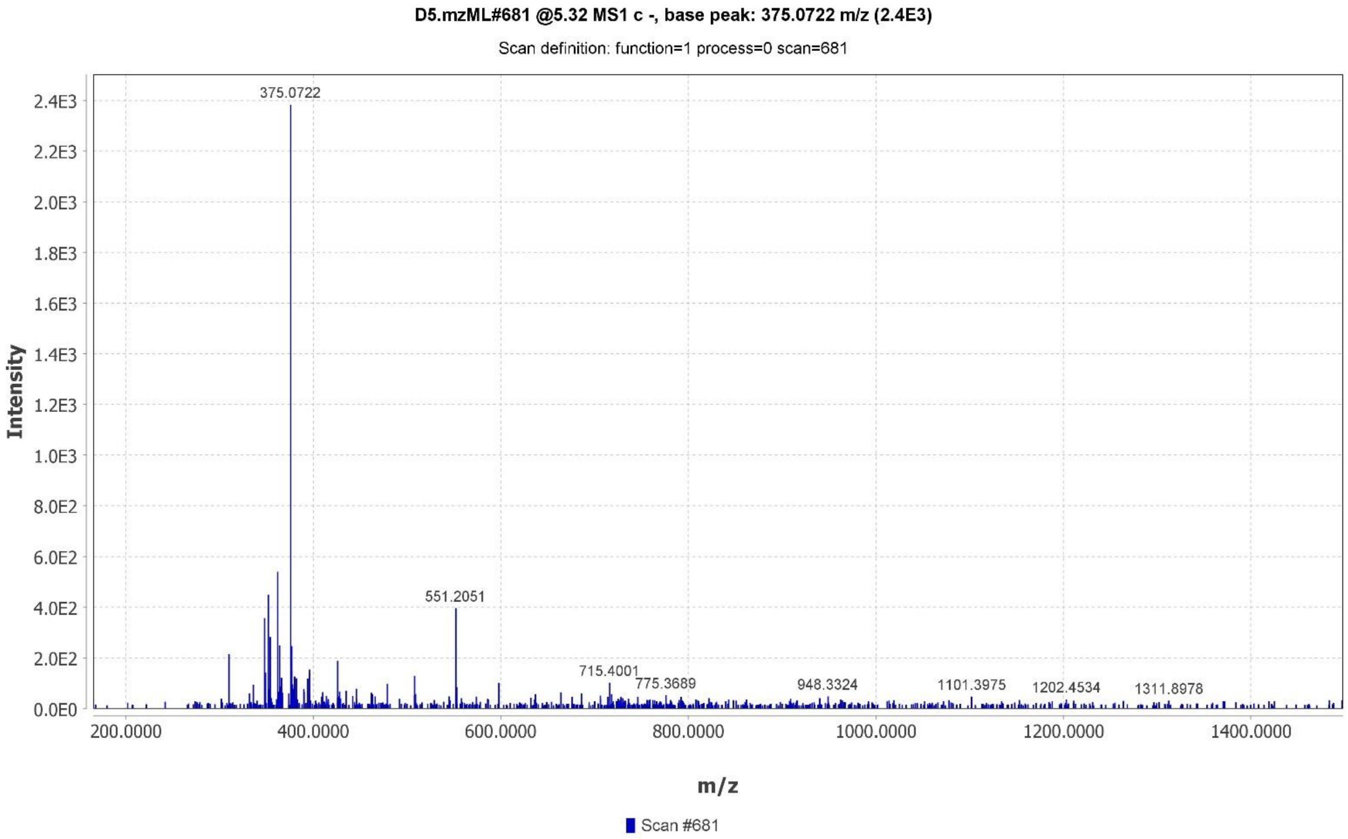

Figure S12. ESIMS spectrum for 5,7,4',5' -tetrahydroxy-3,6,2’-trimethoxyflavone (**2**)

1.3.3. Hautriwaic acid lactone (3)

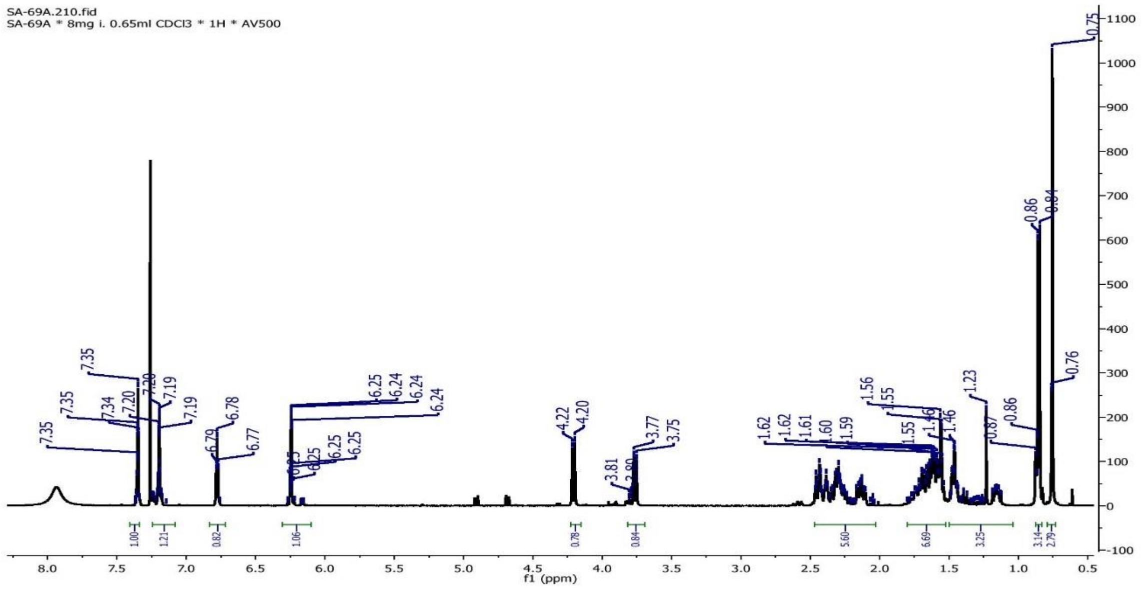

Figure S13. The ^{1}H- NMR spectrum of hautriwaic acid lactone (**3**) observed at 500 MHz in $CDCl_3$ solution at 25 °C. Assignment is provided in Table S3.

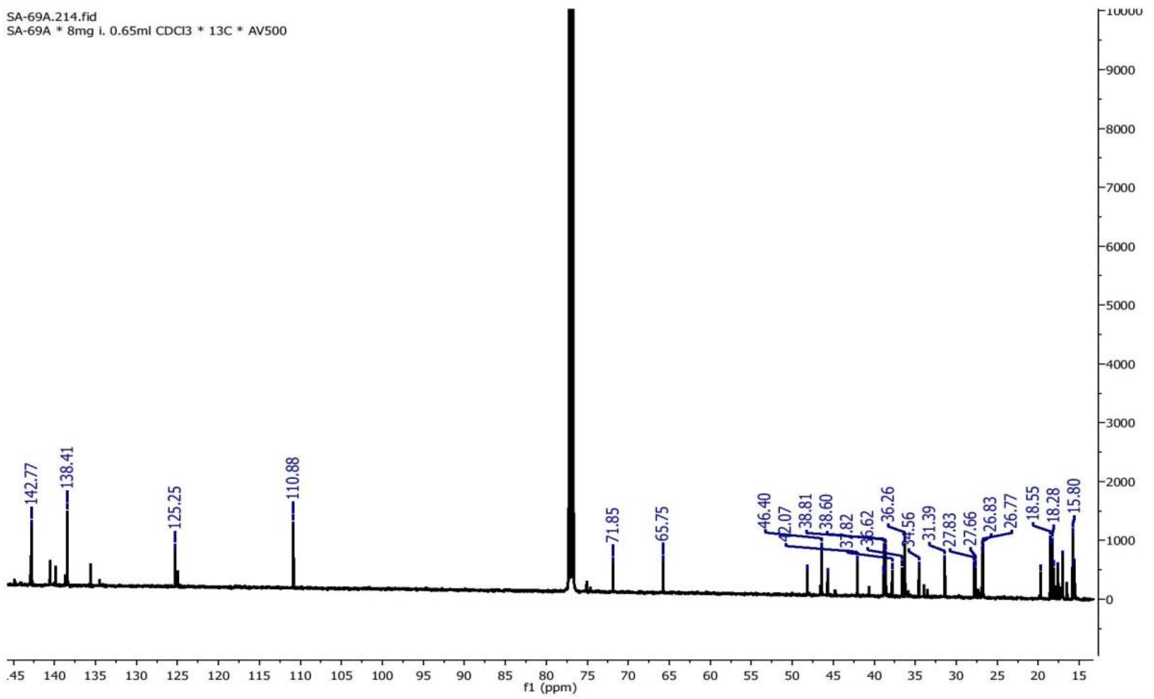

Figure S14. ^{13}C- NMR spectrum of hautriwaic acid lactone (**3**) observed at 500 MHz. in $CDCl_3$ solution at 25 °C. Assignment is provided in Table S3.

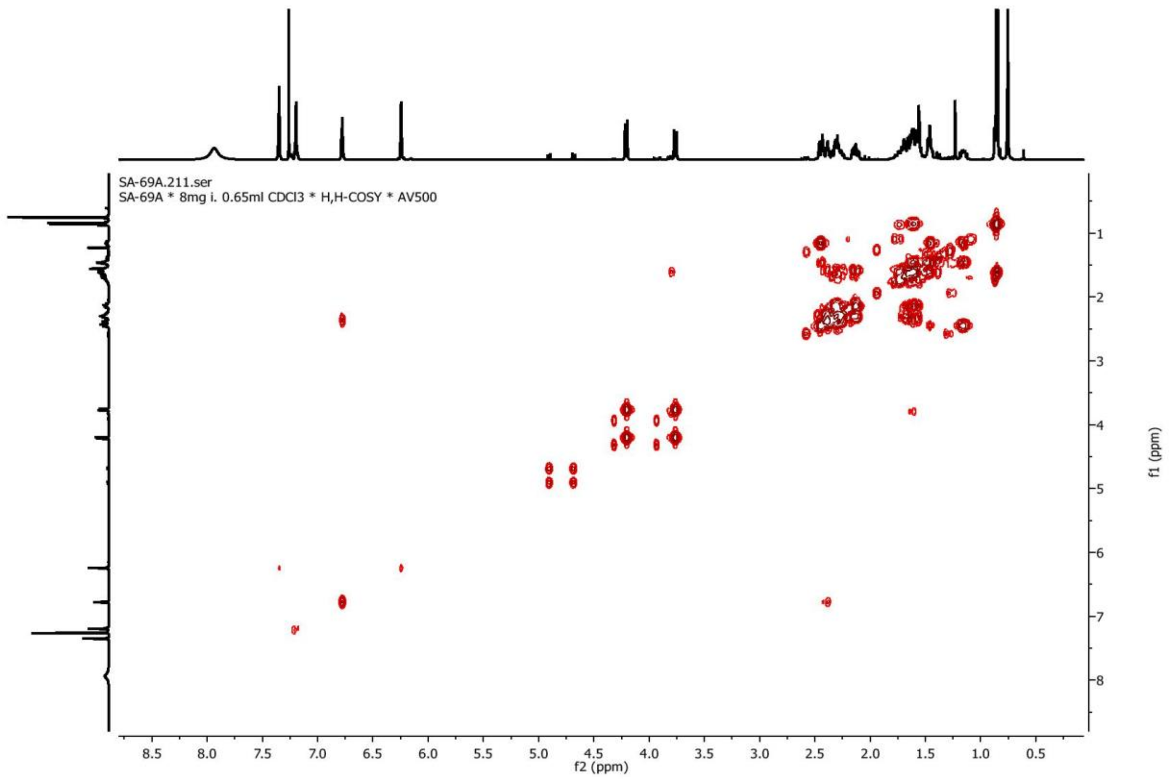

Figure S15. The ^{1}H- ^{1}H COSY- NMR spectrum of hautriwaic acid lactone (**3**) observed at 500 MHz in $CDCl_3$ solution at 25 °C.

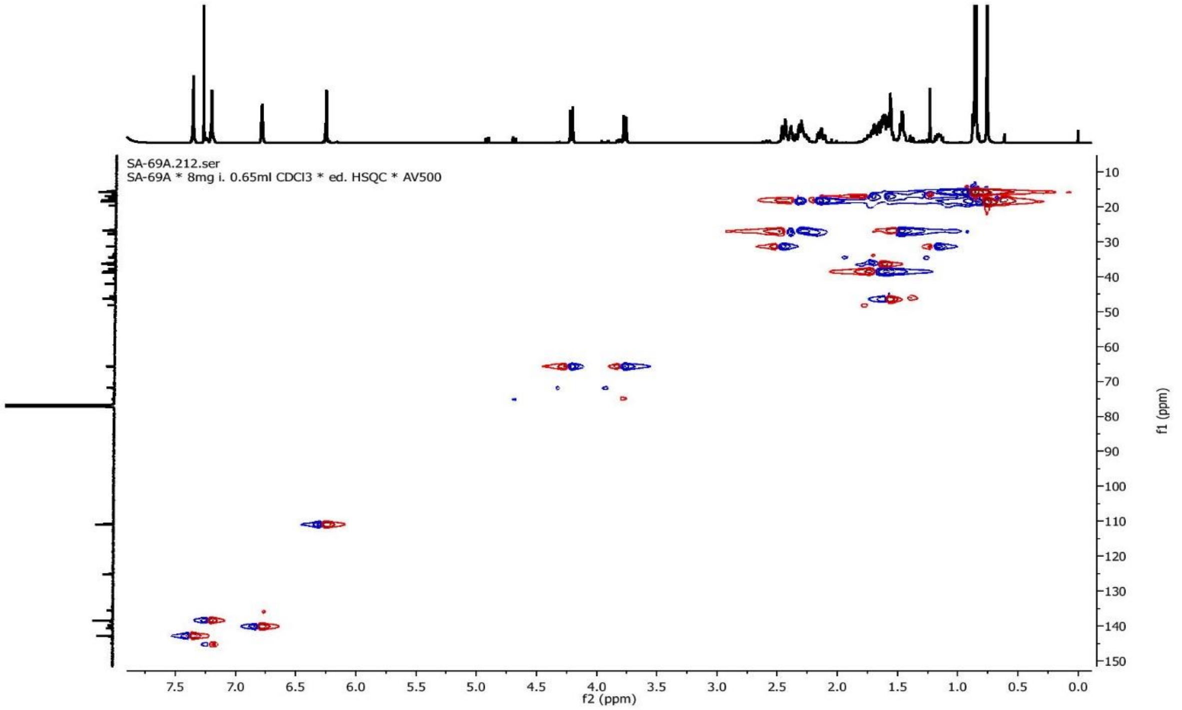

Figure S16. The ^{1}H- ^{13}C- HSQS NMR spectrum of hautriwaic acid lactone (**3**) observed at 500 MHz in $CDCl_3$ solution at 25 °C.

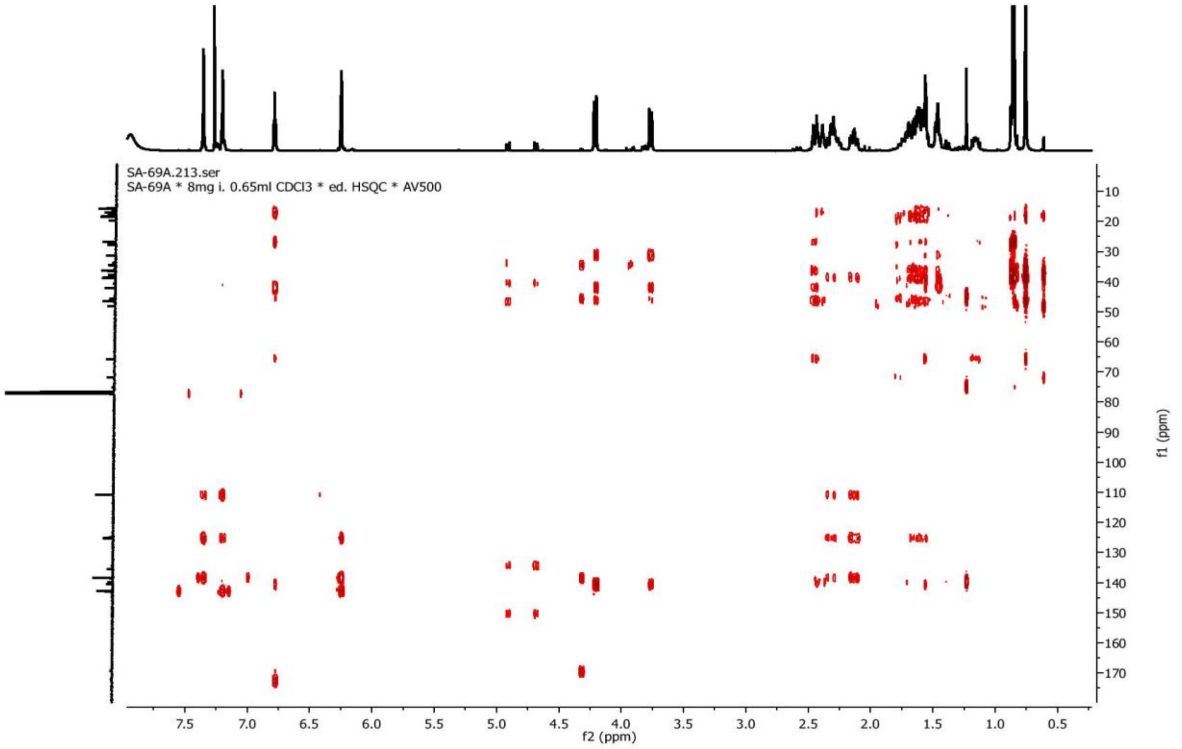

Figure S17. The ^{1}H- ^{13}C- HMBC NMR spectrum of hautriwaic acid lactone (**3**) observed at 500 MHz in $CDCl_3$ solution at 25 °C. Assignment is provided in Table S3.

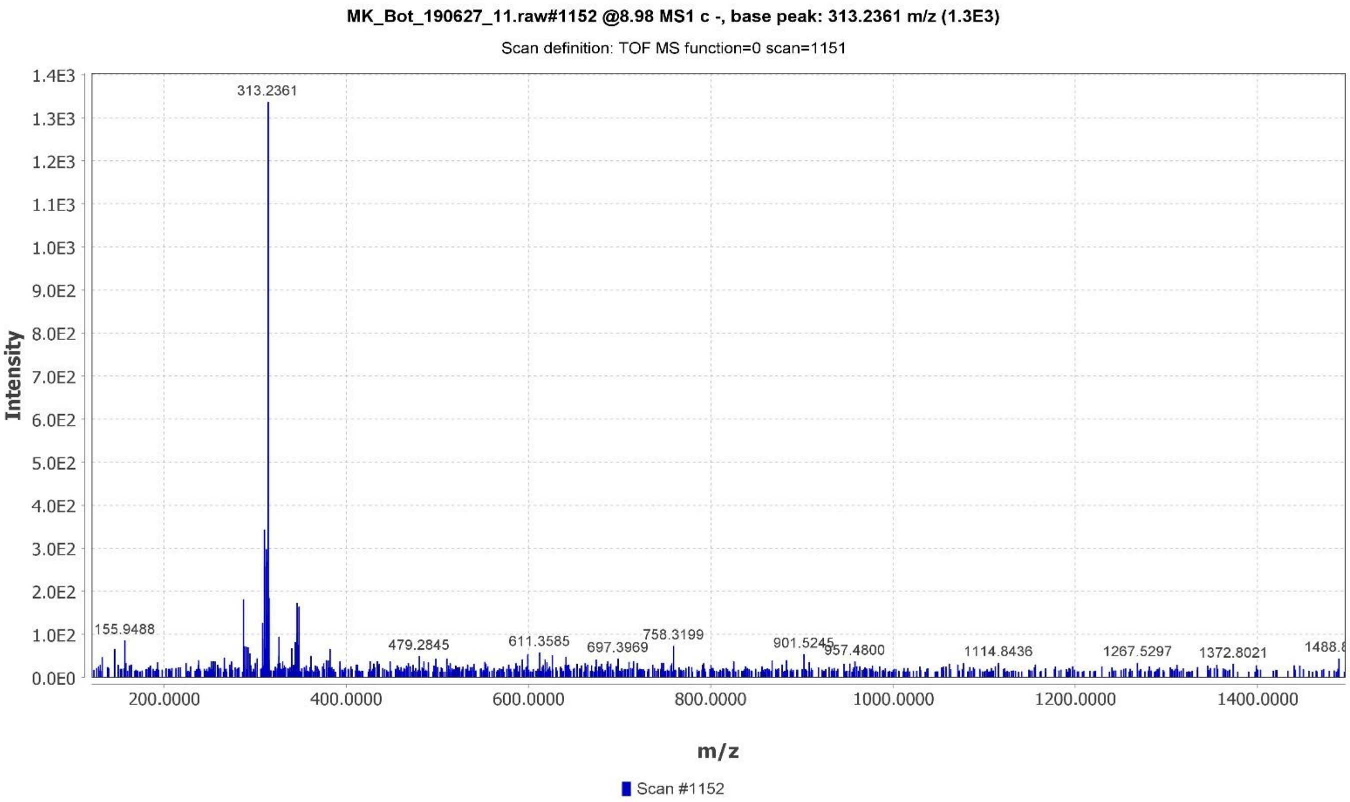

Figure S18. ESIMS for hautriwaic acid lactone (**3**)

1.3.4. Catechin (4)

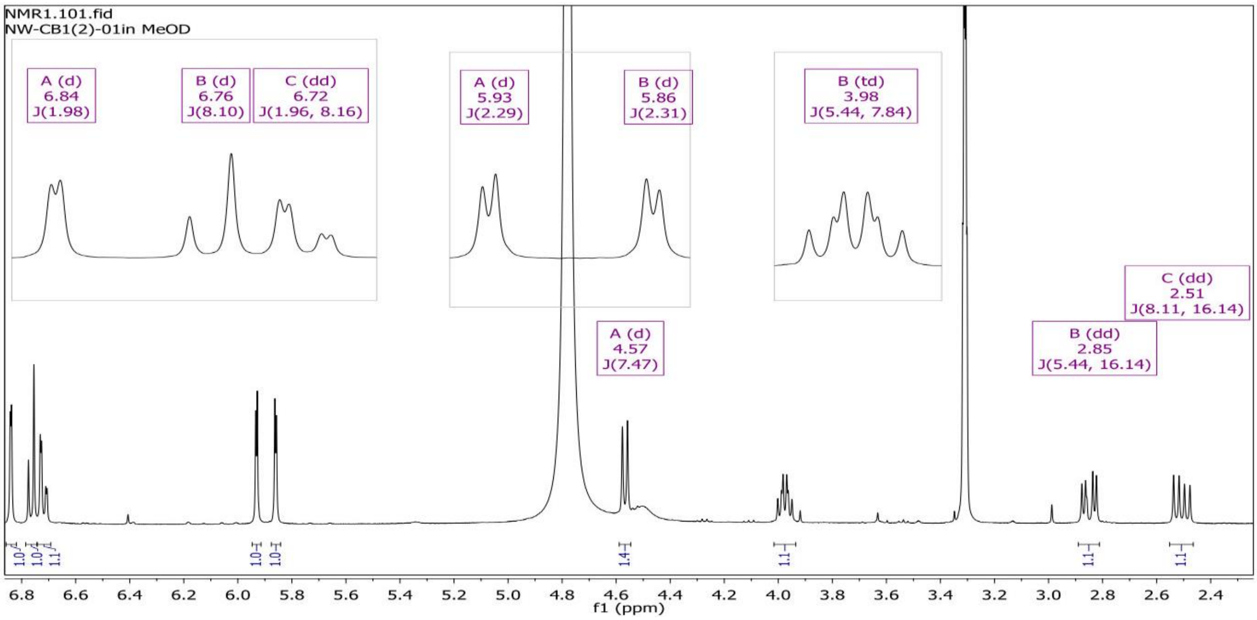

Figure S19. The ^{1}H NMR spectrum of catechin (**4**) observed at 500 MHz in MeOD solution at 25 °C. Assignment is provided in Table S4.

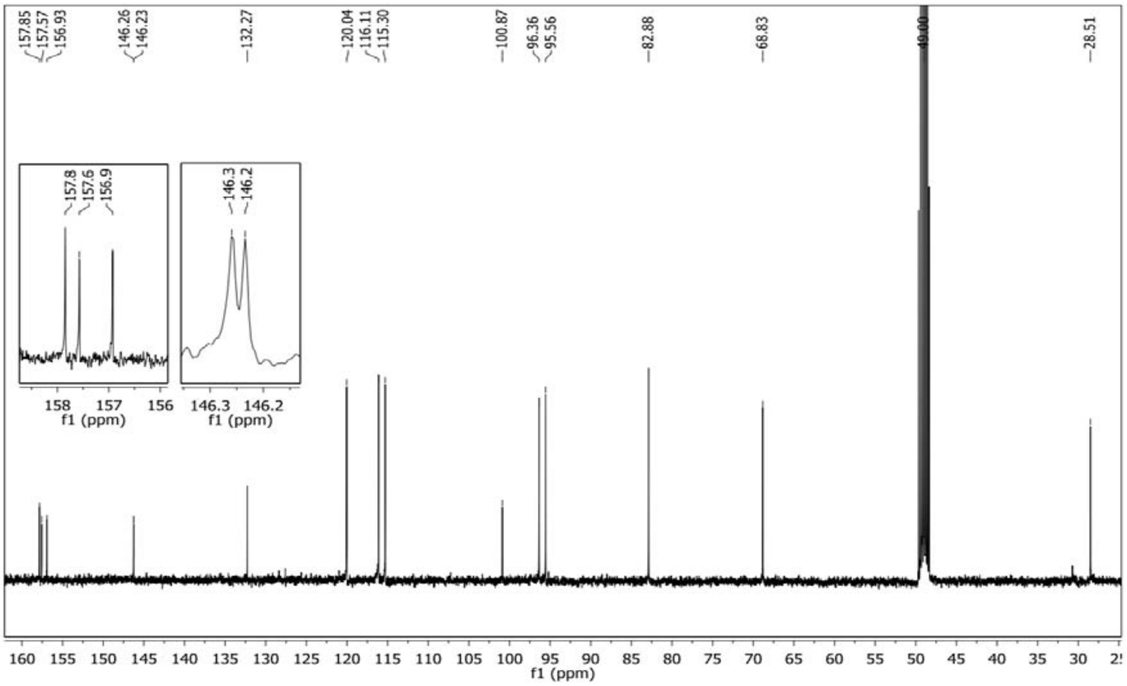

Figure S20. The ^{13}C NMR spectrum of catechin (**4**) observed at 500 MHz in MeOD solution at 25 °C. Assignment is provided in Table S4.

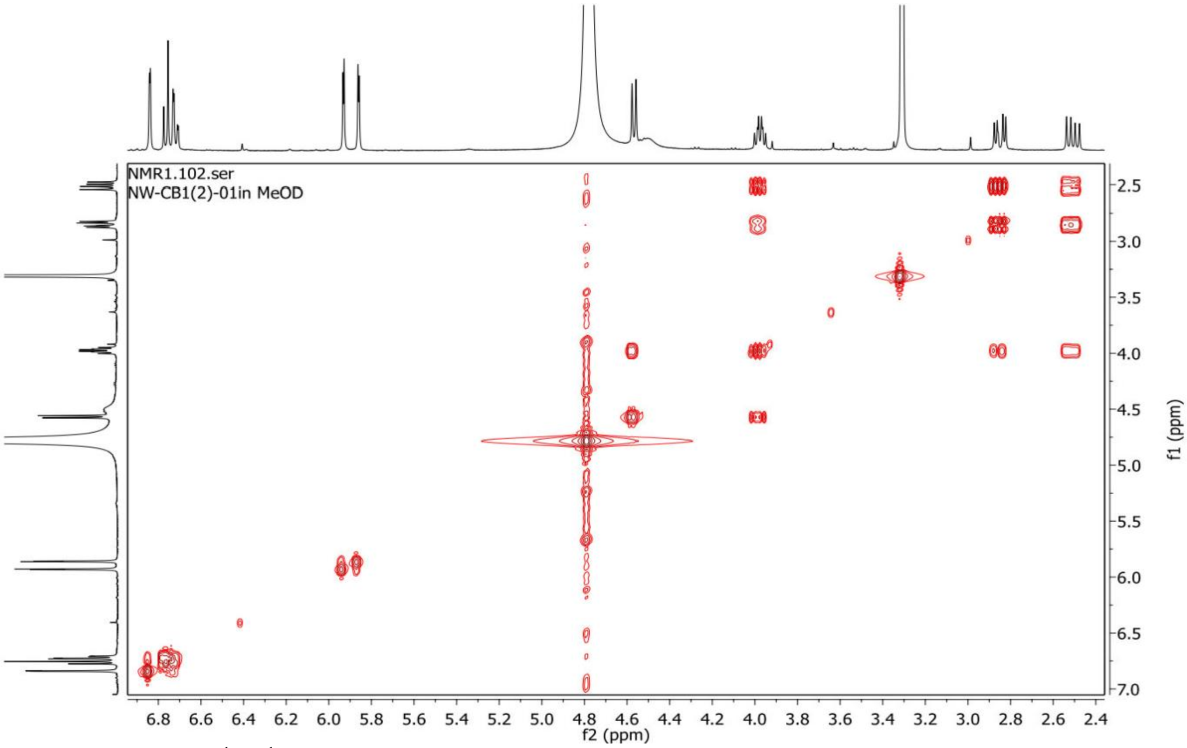

Figure S21. The ^{1}H-^{1}H COSY spectrum of catechin (**4**) observed at 500 MHz in MeOD solution at 25 °C.

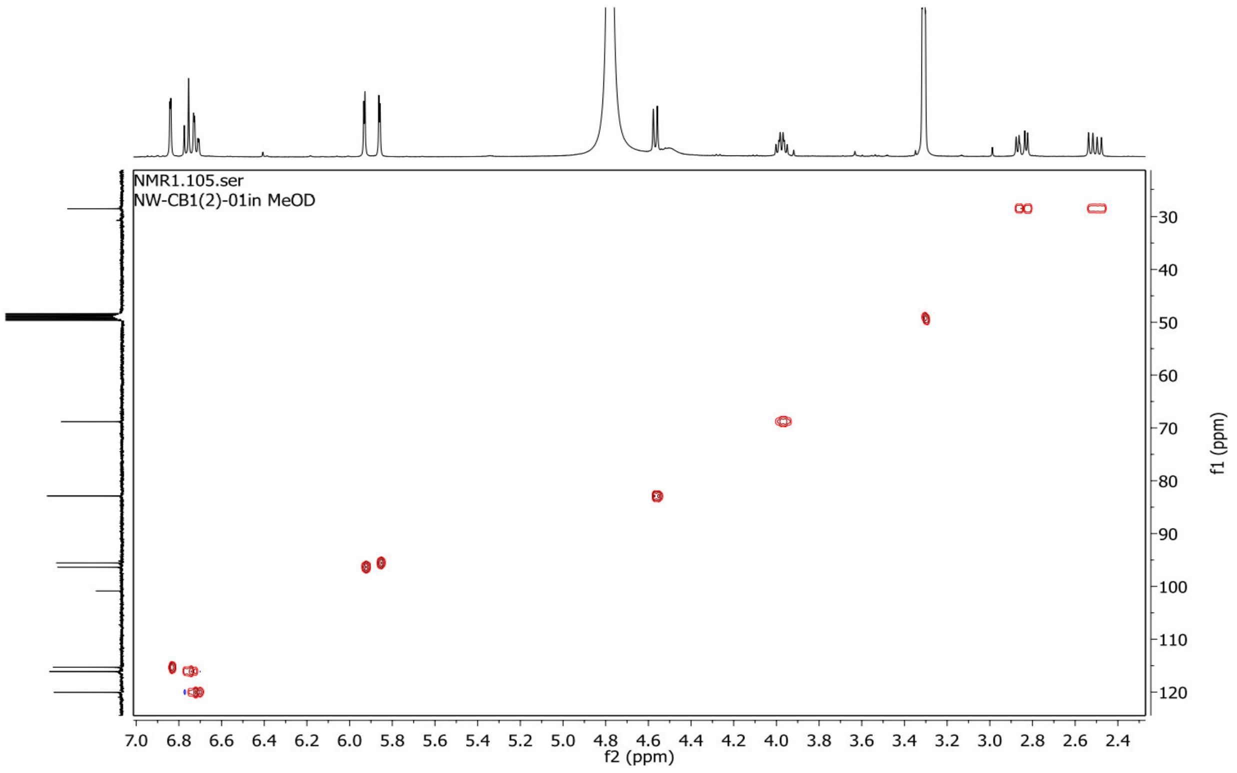

Figure S22. The ^{1}H-^{13}C HSQC NMR spectrum of catechin (**4**) observed at 500 MHz in MeOD solution at 25 °C.

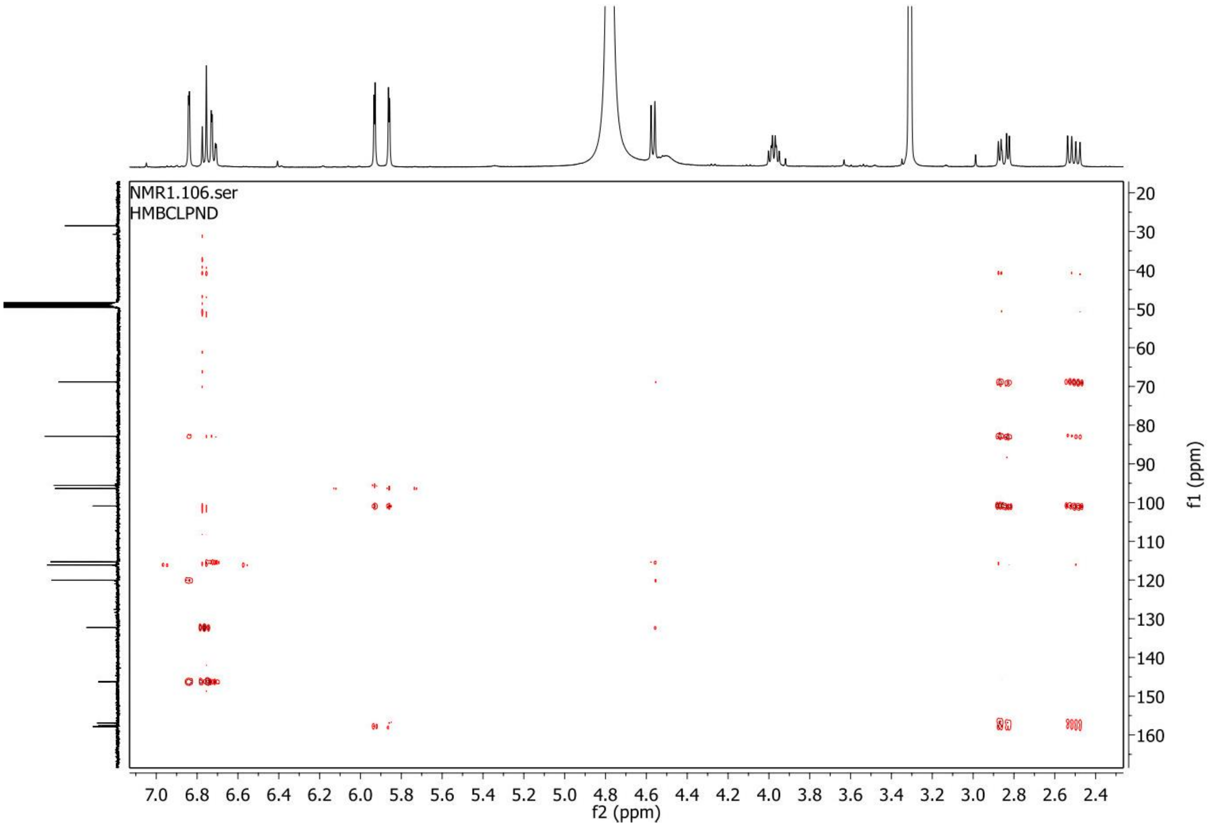

Figure S23. The ^{1}H-^{13}C HMBC NMR spectrum of catechin (**4**) observed at 500 and 125 MHz in MeOD solution at 25 °C. Assignment is given in Table S4

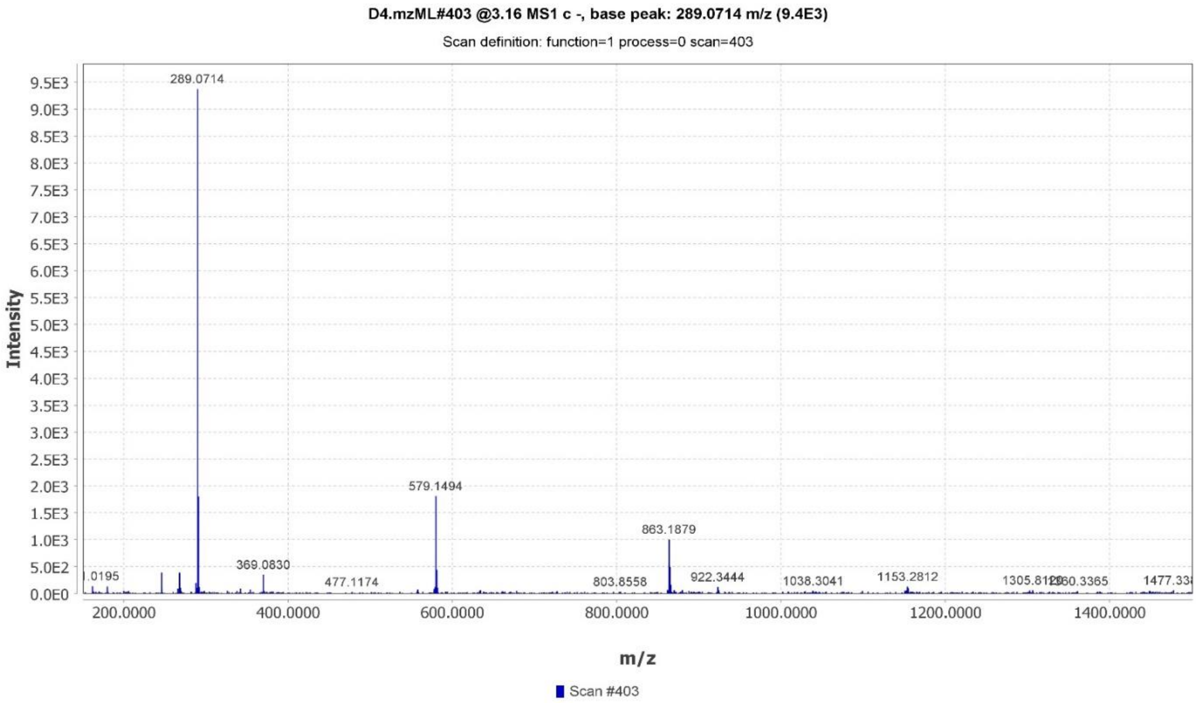

Figure S24. ESIMS for catechin (**4**).

1.3.Random permutation and k-fold cross-validation of discriminant chemicals for Kenyan *D. viscosa*

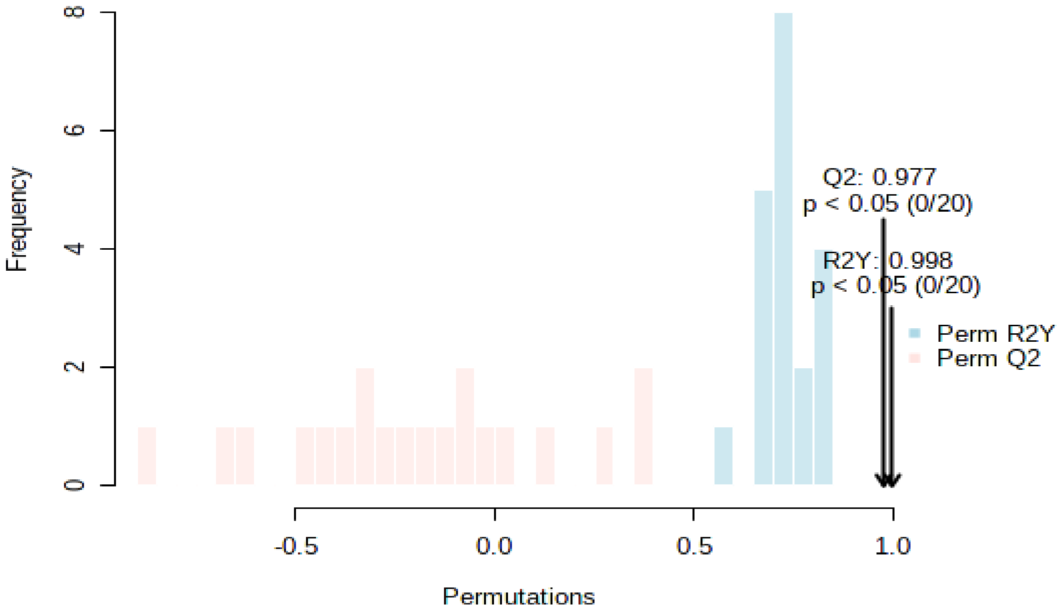

Figure S25. Random permutation of discriminant chemicals for the five populations of *D. viscosa* in Kenya

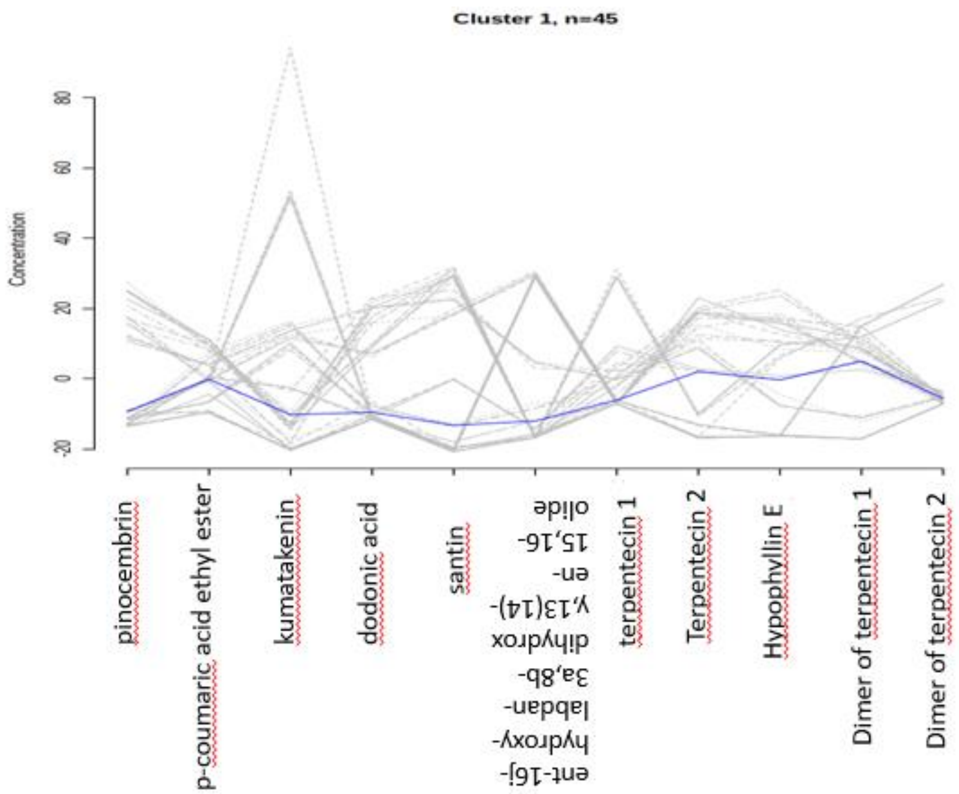

Figure S26. k-fold cross-validation of discriminant chemicals for the five populations of *D. viscosa* in Kenya

References

1. Omosa, L.K.; Amugune, B.; Ndunda, B.; Milugo, T.K.; Heydenreich, M.; Yenesew, A.; Midiwo, J.O. Antimicrobial flavonoids and diterpenoids from *Dodonaea angustifolia*. South African J Bot [Internet]. 2014;91:58–62. Available from: http://dx.doi.org/10.1016/j.sajb.2013.11.012
2. Sachdev, K.; Kulshreshtha, D.K. Dodonic Acid, A New Diterpenoid from *Dodonaea viscosa.* Planta Medica. 1984;50: 448-449.
3. Yu, S.; Fang, N.; Mabry, T.J. Flavonoids from *Gymnosperma glutinosum*. Phytochemistry. 1988;27(1):171–7.
4. Naidoo, R.; Patel, M.; Gulube, Z.; Fenyvesi, I. Inhibitory activity of *Dodonaea viscosa* var. angustifolia extract against Streptococcus mutans and its biofilm. J Ethnopharmacol [Internet]. 2012;144(1):171–4. Available from: http://dx.doi.org/10.1016/j.jep.2012.08.045
